# Novel Concomitant Variations of the Tibialis Anterior and Extensor Hallucis Longus: Clinical Implications of Accessory Tendons

**DOI:** 10.7759/cureus.107223

**Published:** 2026-04-17

**Authors:** Jake Dourdourekas, Julia Petti, Audrey Pike, Kaden L Wilson, Jay M Bauman

**Affiliations:** 1 Department of Anatomical Sciences, Saint Louis University School of Medicine, St. Louis, USA

**Keywords:** accessory muscle tendon, anatomical variation, anterior compartment, extensor hallucis longus (ehl) tendon, tibialis anterior tendon

## Abstract

The tibialis anterior (TA) and extensor hallucis longus (EHL) muscles are critical dorsiflexors of the foot and essential for gait stability. During a routine cadaveric dissection, a novel combination of anatomical variations involving the TA and EHL was discovered bilaterally in a 90-year-old male donor. Two accessory tendinous slips emerged from the TA primary tendon, one inserting into the proximal phalanx of the hallux and the other onto the shaft of the first metatarsal. Concurrently, an accessory EHL tendon arising from the EHL muscle belly merged with the tendon of the extensor hallucis brevis (EHB) to form a conjoined tendon inserting on the proximal phalanx of the hallux. A literature review was then used to categorize these findings according to established classification systems and reported prevalences. Knowledge of this concomitant variation is useful in preoperative planning and treatment of pathologies such as tendon ruptures and hallux valgus, the development of which has been linked to variations in the EHL and TA similar to those in the case presented here.

## Introduction

The tibialis anterior (TA) and extensor hallucis longus (EHL) muscles are two of the four muscles located in the anterior compartment of the leg. The TA is characterized as inserting on the inferomedial surfaces of the medial cuneiform and the base of the first metatarsal, and the EHL is generally described as inserting on the dorsal surface of the distal phalanx of the hallux. While both muscles contribute to dorsiflexion of the foot, the TA is also responsible for foot inversion, and the EHL extends the first digit [1].

Variations in the distal insertions of both the TA and the EHL have been widely reported in the literature. The TA has shown considerable variation in its relative insertion size on the medial cuneiform and first metatarsal, with some studies showing a large attachment to the medial cuneiform and small attachments to the first metatarsal, and other studies demonstrating the reverse [2-5]. Other documented TA variations include additional tendinous slips extending to the shaft or distal part of the first metatarsal [3,4], or a sole attachment to the medial cuneiform or first metatarsal [2,4,6].

The EHL has frequently been reported to have one or more accessory tendons that insert onto a variety of sites on the dorsal surface of the foot. A common variation is an accessory tendon arising from the EHL muscle belly and inserting on the proximal phalanx of the hallux medially, distally, or directly into the extensor hallucis brevis (EHB) insertion. Reported variations are diverse; notably, trifurcations of the primary EHL tendon have been observed with attachments extending into the metatarsophalangeal joint capsule [7,8].

These variant anomalies are clinically relevant to the treatment of various foot and ankle deformities. Because the TA and EHL are frequently harvested for tendon transfers to treat hallux valgus (HV), hallux varus, and congenital talipes equinovarus, unexpected tendon anomalies such as accessory slips can alter the expected biomechanical outcome of the procedure [9-11]. Furthermore, the literature remains divided on whether specific variant patterns, such as accessory EHL tendons or isolated TA insertions, serve as predisposing factors for the development of HV [12,13].

This paper aims to provide insight into concomitant TA and EHL variations by reporting and classifying a novel concomitant TA and EHL variation pattern. Characterization of coexisting variations is necessary to ensure optimal operative strategies and patient outcomes in conditions involving the dorsal foot.

## Case presentation

A novel and complex set of concomitant variations was observed in the leg during a routine dissection as part of a medical school gross anatomy course. The body donor was a 90-year-old male. All donors were obtained through the Saint Louis University Gift Body Program of the Center for Anatomical Science and Education (CASE), with signed informed consent from the donors. The CASE Gift Body Program follows all rules set forth by the Uniform Anatomical Gift Act. While these variations were observed bilaterally, this report focuses on the left leg, as the tendons of the right leg were transected during a subsequent dissection.

**Figure 1:**
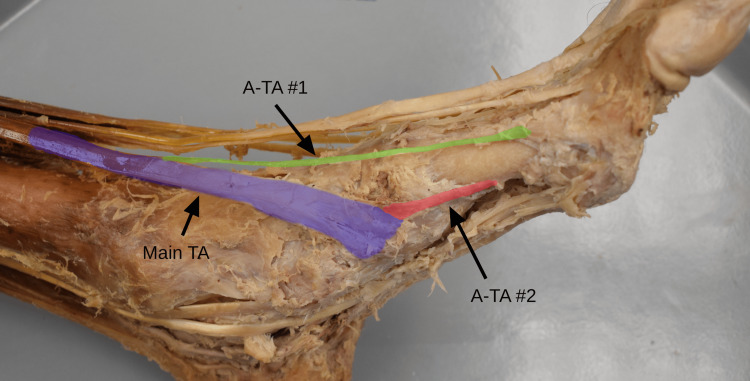
Accessory tendinous slips of the TA Anteromedial view of the left foot demonstrating two accessory tendinous slips of the TA. Main TA tendon (purple), first accessory slip (A-TA #1, green), second accessory slip (A-TA #2, red). TA, tibialis anterior

The TA tendon featured a typical primary attachment to the medial cuneiform and first metatarsal (Figure 1). However, two accessory tendinous slips emerged from the primary TA tendon: one inserting on the dorsal portion of the proximal phalanx of the hallux and a second inserting on the medial shaft of the first metatarsal.

**Figure 2:**
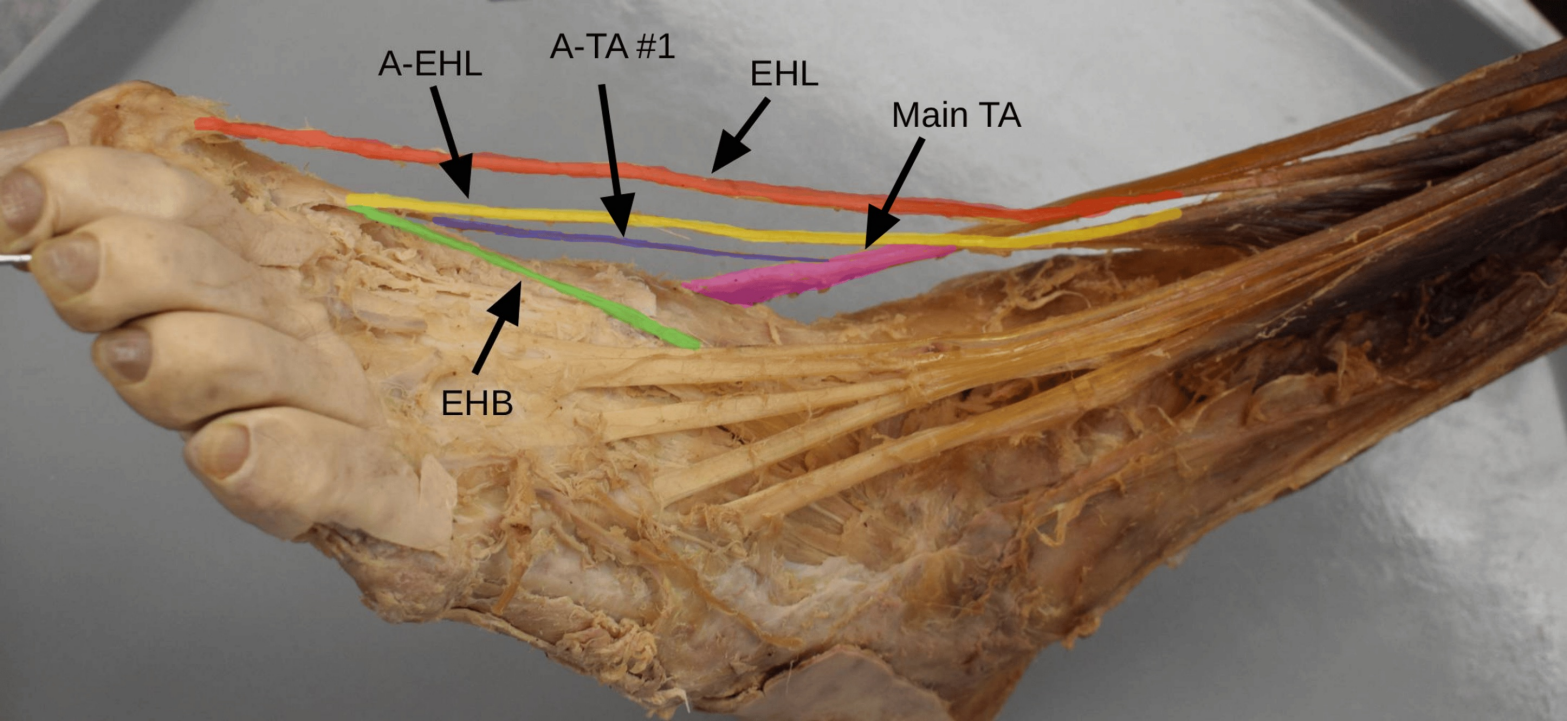
Anterolateral view of the left foot showing accessory EHL and TA tendon variations Main EHL tendon (red), A-EHL (yellow), EHB (green), main TA (pink), first accessory slip of TA (A-TA #1, purple). A-EHL, accessory tendon of extensor hallucis longus; EHB, extensor hallucis brevis; EHL, extensor hallucis longus; TA, tibialis anterior

The primary EHL tendon pursues a distal course to its insertion at the base of the distal phalanx of the hallux (Figure 2). Concurrently, the EHL presented an accessory tendon originating from a separate muscle belly that fused with the tendon of EHB. This conjoined tendon then inserted on the proximal phalanx of the hallux (Figure 2).

## Discussion

A literature search was conducted using the PubMed/MEDLINE and Google Scholar databases to identify studies describing anatomical variations of the TA. The search strategy employed a combination of Medical Subject Headings (MeSH) and specific Title/Abstract keywords, including ('tibialis anterior tendon'[Title/Abstract] OR 'anterior tibial tendon'[Title/Abstract] OR TAT[Title/Abstract]) AND ('classification scheme'[Title/Abstract] OR 'classification system'[Title/Abstract] OR 'grading system'[Title/Abstract] OR 'typology'[Title/Abstract] OR 'morphological variant'[Title/Abstract] OR 'new classification'[Title/Abstract] OR classification[MeSH] OR 'Anatomic Variation'[MeSH]). Studies were included for analysis if they proposed or utilized a classification scheme based on primary cadaveric or ultrasonographic data. This search process revealed six studies [2-6,13] that currently define the morphological landscape of TA insertions, the findings of which are categorized in Table 1.

**Table 1:** Summary of reported prevalences for TA insertion patterns across cadaveric anatomical studies The dash “-” indicates that the specific morphological variation was not observed or recorded within that particular study. FMT, first metatarsal; MC, medial cuneiform; TA, tibialis anterior

Study	N (legs)	Type 1: One tendon attaching to the MC and FMT	Type 2: One tendon inserting on the MC or FMT	Type 3: Primary tendon that attaches to MC and FMT with supernumerary slips
Brenner [2]	156	96.2%	3.2%	-
Musiał [3]	122	95.9%	-	4.1%
Olewnik et al. [4]	100	66%	32%	2%
Willegger et al. [5]	41	100%	-	-
Arthornthurasook and Gaew-Im [6]	44	84.1%	15.9%	-
Karauda et al. [13]	100	40%	60%	-

Due to the lack of a universal nomenclature across the identified literature, a unified three-tier classification system was developed for this review to facilitate qualitative comparison. Studies that did not originally utilize a Type 1-3 scheme (e.g., [5]) were mapped to this study’s criteria based on their reported anatomical descriptions of the distal attachment sites. Studies containing more than three classification tiers (e.g., [3,4]) were consolidated into the present Type 1-3 framework. Subvariants were grouped based on their primary insertion morphology: single bifurcated insertions were categorized as Type 1, isolated single-site insertions as Type 2, and any presence of accessory or supernumerary slips, regardless of specific distal termination, was grouped as Type 3.

The anatomical variation observed in the present study is categorized as Type 3 (primary tendon attaching to the MC and FMT with supernumerary slips), consistent with the classification scheme utilized in Table 1. Morphologically similar variants have been documented in previous literature, though under differing nomenclature; specifically, these were reported as “Type III” by Musial [3] and “Type IV” by Olewnik et al. [4]. The prevalence of these variants was documented at 4.1% (N = 122 limbs) and 2% (N = 100 limbs), respectively. Notably, these studies represent the only reports in the literature review to describe supernumerary slips originating from the primary TA tendon, as other classification schemes focus exclusively on bifurcation or isolation of the main insertion.

A parallel literature search was conducted using the PubMed/MEDLINE and Google Scholar databases to identify studies describing anatomical variations of the EHL. The search strategy employed a combination of MeSH and specific Title/Abstract keywords, including ('extensor hallucis longus'[Title/Abstract] OR 'EHL'[Title/Abstract] OR 'extensor hallucis longus tendon'[Title/Abstract]) AND ('classification scheme'[Title/Abstract] OR 'classification system'[Title/Abstract] OR 'grading system'[Title/Abstract] OR 'typology'[Title/Abstract] OR 'morphological variant'[Title/Abstract] OR 'accessory tendon'[Title/Abstract] OR 'new classification'[Title/Abstract] OR classification[MeSH] OR 'Anatomic Variation'[MeSH]). Through this review, two primary classification schemes for EHL variations have been identified (Table 2). The reported prevalences of EHL variations were classified by the number of accessory tendons and their insertion patterns.

**Table 2:** Comparative prevalences of accessory EHL tendon configurations and insertion sites as reported by Al-saggaf and Olewnik et al. The dash “-” indicates that the specific morphological variation was not observed or recorded within that particular study. EHB, extensor hallucis brevis; EHL, extensor hallucis longus

Number of accessory tendons	Insertion configuration	Al-saggaf [7]	Olewnik et al. [8]
None	Single tendon inserting on the distal phalanx of the hallux	65.00%	57.50%
One (main tendon inserting on the extensor hood of the distal phalanx of the hallux)	Independent insertion into the proximal phalanx of the hallux	23.30%	34.70%
One (main tendon inserting on the extensor hood of the distal phalanx of the hallux)	Joining the EHB termination at the proximal phalanx	5.00%	-
One (main tendon inserting on the extensor hood of the distal phalanx of the hallux)	Merging with the middle of the EHB, forming a conjoined tendon that inserts on the proximal phalanx of the hallux	3.30%	-
One (main tendon inserting on the extensor hood of the distal phalanx of the hallux)	Inserting onto the dorsal aspect of the first metatarsal	-	5.70%
Two (main tendons inserting on the extensor hood of the distal phalanx of the hallux)	Splitting from the main tendon and inserting into the hallux	8.30%	1.90%

Within the two classification schemes, the current variation of the EHL most closely aligns with the “One accessory tendon” category, specifically the configuration in which the accessory tendon merged with the EHB and that conjoined tendon inserted on the proximal phalanx of the hallux. This variation was previously reported to occur in 3.3% of 60 examined lower limbs by Al-saggaf [7]. Although Olewnik et al. did not document this precise anatomical form of an EHL accessory tendon merging with the EHB, they reported several closely related variants in which accessory slips of the EHL inserted on the proximal phalanx of the hallux independently [8].

Additionally, this variation resembled a form of the EHL known as the extensor primi internodii hallucis of Wood (EPIH). The EPIH represents an accessory slip that inserts onto the dorsal aspect of the base of the proximal phalanx of the hallux, often inserting near or merging with the distal attachment of the EHB [14]. Its frequency is debated, as some studies treat this as a rare anomaly, while others suggest it is relatively common.

A significant finding in this study is the presence of concomitant variations involving both the EHL and TA. While individual variations are well documented in the literature, they are almost exclusively reported independently. Reports of concurrent variations in the anterior compartment are sparse; for example, Patel et al. [12] described a complex merger of accessory EHL and accessory EHB tendons. However, the present case represents the first documented instance of these specific variations of the TA and EHL occurring together.

These anatomical variations arise during the formation of the appendicular skeleton in the embryonic period. The synovial joint is formed from interzonal mesenchyme between the primordia of bones, and HOX genes regulate limb patterning in conjunction with FGF signaling from the apical ectodermal ridge. Subsequently, the common extensor muscle mass of the anterior compartment of the leg undergoes fragmentation, migration, and rearrangement. Discrete muscles form from proximal to distal, while tendon development occurs in the opposite direction. It is likely that subtle variations in signaling and gene expression during these events result in phenotypic differences [15].

Comprehensive awareness of EHL and TA anatomy is vital for successful operations in the dorsal foot. Surgical treatment of several common foot deformities utilizes TA or EHL tendons, including correction of HV, hallux varus, and equinovarus [9,16,17]. For instance, transferring the TA insertion to the lateral cuneiform is a proven technique for clubfoot correction [16]. The close functional relationship between these muscles allows for their use as reciprocal autografts, utilizing one tendon to repair a rupture in the other. EHL tendon transfer is a common method for TA rupture repair [18].

Consequently, the presence of accessory tendons, as in the present case, increases clinical complexity. The efficacy of these procedures depends heavily on the surgeon’s ability to navigate the patient’s specific insertion anatomy and recognize accessory structures that may complicate the surgical field. Additionally, the presence of an accessory tendon can provide an advantageous source for tendon transfer during repair. The clinical utility of these accessory tendons is reported in a case report where an accessory EHL tendon was successfully utilized to repair a primary EHL injury. This method restored function without the donor-site morbidity associated with remote autografts or the rejection risks of allografts [19]. However, further series are needed to determine whether this approach can be broadly replicated.

A primary point of clinical contention is the role that variations in the distal insertions of these muscles play in the development of HV. The literature regarding the EHL is currently divided; while several cadaveric studies suggest a higher prevalence of HV in feet possessing an accessory EHL tendon [7,10,20], others found no correlation [17]. Similarly, it is debated whether specific TA insertion types are linked to HV. Brenner found no correlation between the two [2]; however, more recent data suggest that TAs that insert solely into the medial cuneiform or have increased tendon diameter are significantly associated with the deformity [11]. While mechanistic links to explain these relationships have been proposed, they remain speculative, and biomechanical studies are required to quantify the influence of these variations on HV and, more generally, on foot stability.

## Conclusions

Variations in the EHL and TA are complex and clinically significant findings. The discovery of concomitant variations in the same limb highlights the need for a “tailored” surgical approach. While surgeons may view accessory EHL tendons as valuable tools for autografting and reconstruction, they must also recognize that these tendons and TA variations may contribute to foot deformities. Recognition of TA and EHL variations allows practitioners to refine surgical approaches to account for each patient’s unique anatomy.
